# Genome-Wide Identification and Functional Verification of Core *Berberine Bridge-like Enzyme* Genes for Benzylisoquinoline Alkaloid Biosynthesis in *Corydalis saxicola* Bunting

**DOI:** 10.3390/ijms27156708

**Published:** 2026-07-27

**Authors:** Liang Kang, Han Liu, Dan Zhu, Cui Li, Ming Lei, Zhanjiang Zhang

**Affiliations:** 1 Guangxi Key Laboratory of Medicinal Resources Protection and Genetic Improvement, Guangxi Botanical Garden of Medicinal Plants, Nanning 530023, China; 17736623451@163.com (L.K.); gmhg_lh@163.com (H.L.); licuicui941@163.com (C.L.); 2 School of Pharmacy, Guangxi Medical University, Nanning 530021, China; zhudan@stu.gxmu.edu.cn; 3 National Traditional Chinese Medicine (TCM) Inheritance and Innovation Center, Nanning 530023, China; 4 National Engineering Research Center for Southwest Endangered Medicinal Materials Resources Development, Nanning 530023, China

**Keywords:** *Corydalis saxicola* bunting, *Berberine bridge-like enzyme* gene family, substrate specificity, dehydrocavidine biosynthesis, in vitro enzymatic assay

## Abstract

Benzylisoquinoline alkaloids (BIAs) are significant defensive and pharmaceutical metabolites found in medicinal plants of the Papaveraceae family. Among these, dehydrocavidine is a distinctive bioactive constituent of *Corydalis saxicola* Bunting, an endangered medicinal plant endemic to karst habitats, known for its definite hepatoprotective, anti-inflammatory, and antiviral pharmacological properties. Berberine bridge enzyme-like (*BBEL*) proteins serve as key rate-limiting enzymes catalyzing core skeletal oxidation in BIA biosynthesis. However, their functions in *C. saxicola* remain uncharacterized. In this study, we performed a comprehensive functional genomic analysis of the *BBEL* family in *C. saxicola* (*CsBBELs*). A total of 22 *CsBBEL* members were identified from the genome of *C. saxicola*, all of which possess the conserved FAD-binding domain and BBE functional domain characteristic of the *BBEL* family. Phylogenetic analysis revealed that the *CsBBEL* family formed three clades closely clustered with homologs from related Papaveraceae species, indicating high conservation. Expression profiles demonstrated significant organ specificity and Ca^2+^ stress response of the *CsBBEL* genes, with *CsBBEL3*, *CsBBEL16*, and *CsBBEL17* being particularly highly expressed in roots. Protein structure prediction and molecular docking suggested that these candidates bind cavidine and tetrahydrocolumbamine. In vitro enzymatic assays confirmed that *CsBBEL3/16/17* specifically catalyzed the four-electron oxidation of cavidine and tetrahydrocolumbamine to produce dehydrocavidine and columbamine, respectively, exhibiting oxidase activity in the BIA biosynthetic pathway. These findings not only contribute to germplasm conservation but also lay a foundation for the synthetic biology-based improvement of *C. saxicola*.

## 1. Introduction

Benzylisoquinoline alkaloids (BIAs) represent a vital class of plant secondary metabolites, integral to the formulation of numerous widely utilized pharmaceuticals, including morphine, codeine, and berberine [1]. Among the *Corydalis* species, protoberberine-type BIAs are the predominant subclass, recognized for their diverse pharmacological activities, such as antibacterial, antitumor, and neuroprotective properties [2]. Dehydrocavidine (DHCA), the principal bioactive component of the karst-endangered plant *Corydalis saxicola* Bunting, is extensively documented for its hepatoprotective, anti-inflammatory, and antiviral properties [3,4]. However, the biosynthetic pathway of DHCA remains largely uncharacterized, posing a significant challenge to the development of metabolic engineering strategies aimed at augmenting DHCA production to satisfy increasing clinical demands.

Within the conserved BIA biosynthetic pathway, berberine bridge enzyme-like *(BBEL*) proteins facilitate the rate-limiting four-electron oxidation of tetrahydroisoquinoline intermediates, resulting in the formation of the characteristic berberine bridge [5]. *BBEL* proteins are FAD-dependent oxidoreductases belonging to the VAO/PCMH superfamily, characterized by a bicovalently bound FAD cofactor and a conserved C-terminal Y/FxN motif essential for catalysis [6]. These proteins operate through a conserved two-step redox cycle, involving hydride transfer to the FAD N5 atom followed by reoxidation via molecular oxygen [7]. Extensive research has elucidated the functions of *BBEL* proteins across BIA-producing plants. In *Coptis chinensis*, the *BBEL* homolog CcTHBO exhibits broad substrate promiscuity, catalyzing the oxidation of various tetrahydroprotoberberine intermediates [8]. In *Macleaya cordata* and *Papaver somniferum*, *BBEL*-related dihydrobenzophenanthridine oxidases (DBOXs) facilitate the final two-electron oxidation steps in sanguinarine biosynthesis [9,10]. In *Corydalis tomentella*, a species-specific tandem duplication event has resulted in an expanded *BBEL* gene cluster, with several CtBBELs verified to oxidize specific protoberberine precursors such as cavidine and stylopine. Phylogenetic analyses further indicate that *BBEL* genes have undergone significant lineage-specific expansion exclusively within Ranunculales [11], with functional divergence primarily driven by tandem gene duplication.

Although individual *BBEL* genes and their homologs in related species have been functionally characterized, the *BBEL* gene family has not been comprehensively analyzed at the genome-wide level in the endangered karst-endemic medicinal plant *C. saxicola*. Ca^2+^, a critical environmental factor of karst habitats, influences *BBEL* expression and subsequent alkaloid accumulation in *C. saxicola* [12]. Furthermore, the biosynthesis of DHCA, the plant’s signature bioactive constituent, is closely linked to *BBEL* catalytic activity [12]. To address this research gap, we performed a systematic genome-wide characterization of the *CsBBEL* gene family. Our specific objectives were to: (1) identify all members of *CsBBEL* family and analyze their evolutionary and structural characteristics; (2) examine their organ-specific expression patterns and responses to exogenous Ca^2+^ treatments; (3) preliminarily verify the catalytic functions of key candidate *CsBBELs* in protoberberine alkaloid biosynthesis, with a focus on DHCA formation. This study constitutes the first comprehensive analysis of the *BBEL* gene family in *C. saxicola*, laying the groundwork for understanding the molecular mechanism underlying DHCA biosynthesis and its regulatory response to calcium, a core environmental determinant of karst habitats.

## 2. Results

### 2.1. Genome-Wide Identification and Physicochemical Characterization of the CsBBELs

Utilizing the hidden Markov models (HMM) of the BBE domain (PF08031) and the FAD-binding oxidase domain (PF01565) as dual probes, a combination of HMMER search and BLASTp homology screening was employed to identify members of the *BBEL* family within the *C. saxicola* genome. A total of 22 *CsBBEL* members were identified and sequentially designated as *CsBBEL1*–*CsBBEL22*, based on their physical positions on individual chromosomes.

The physicochemical properties of 22 *CsBBELs* were systematically examined utilizing the ExPASy ProtParam tool (Table 1). The findings demonstrated that the overall characteristics of this family are highly conserved. The amino acid lengths ranged from 343 to 606 aa, with molecular weights varying between 38.72 and 68.41 kDa. The theoretical isoelectric points (pI) were distributed from 5.21 to 9.58. Among the members, 19 exhibited a pI greater than 7.0, accounting for 86.4% of the total, suggesting that the *CsBBELs* are predominantly composed of alkaline proteins. Instability index analysis revealed that 18 *CsBBELs* had values below 40 and were classified as stable. Only *CsBBEL5*, *CsBBEL8*, *CsBBEL10*, and *CsBBEL13* presented an instability index of 40 or higher and were therefore considered unstable. The aliphatic index of the *CsBBEL* family ranged from 82.96 to 92.96, indicating a high level of thermostability. All members exhibited negative grand average of hydropathicity values (ranging from −0.261 to −0.046), confirming that all *CsBBELs* are hydrophilic.

**Figure 1:**
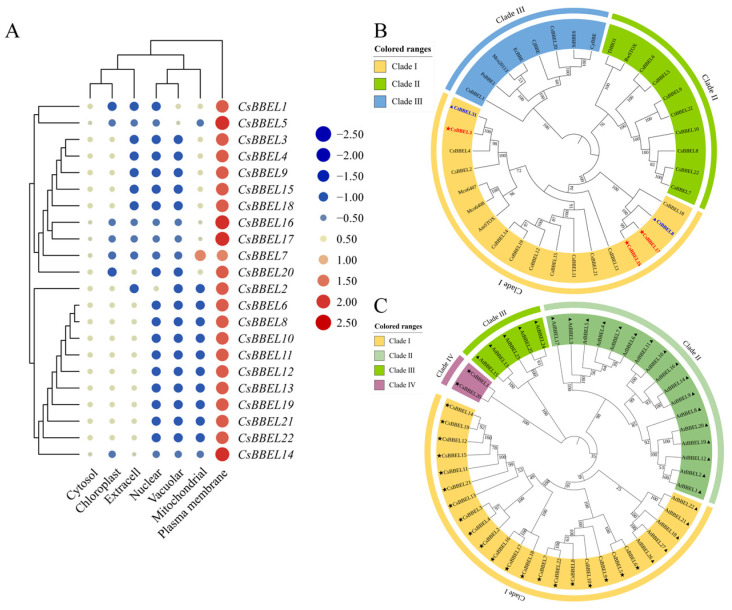
Analysis of subcellular localization and phylogenetic relationships of *CsBBEL* family proteins. (**A**) Heatmap illustrating the subcellular localization prediction scores of 22 *CsBBEL* proteins across various subcellular compartments, with blue indicating low prediction scores and red indicating high scores. (**B**) Phylogenetic tree depicting *CsBBEL* proteins and BBEL/STOX proteins from closely related Papaveraceae species, with bootstrap values presented on the branches, and distinct colors representing major clades. Asterisks indicate proteins from *C. saxicola*, and triangles indicate proteins from *C. tomentella*. (**C**) Phylogenetic tree comparing *CsBBEL* proteins with AtBBEL proteins. Asterisks indicate proteins from *C. saxicola*, and triangles indicate proteins from *Arabidopsis thaliana*.

To explore the functional action sites of *CsBBELs*, subcellular localization prediction was conducted in the present study (Figure 1A). Overall, the plasma membrane emerged as the predominant predicted localization compartment for the *CsBBEL* family, with the majority of members exhibiting moderate to high localization confidence in this region. Notably, *CsBBEL5*, *CsBBEL14*, *CsBBEL16*, and *CsBBEL17* displayed the highest prediction scores for plasma membrane localization, indicating distinct membrane targeting specificity. In comparison, all *CsBBELs* displayed low-to-moderate localization confidence in the cytosol, aligning with the inherent subcellular characteristics of *BBEL*-type oxidoreductases. For other compartments including chloroplast, extracellular space, nucleus, vacuole, and mitochondrion, localization signals were generally weak across the family. Only a small number of members showed appreciable scores in individual compartments; for instance, *CsBBEL7* displayed an additional detectable signal specifically in the mitochondrion. No universally conserved localization pattern for these organelles was shared by all 22 family members. The core candidate genes *CsBBEL3*, *CsBBEL16*, and *CsBBEL17*, identified in subsequent experiments, were consistently predicted to target to the plasma membrane. The plasma membrane localization of these key candidates provide subcellular localization evidence supporting their potential roles in alkaloid biosynthesis.

### 2.2. Phylogenetic Analysis

To elucidate the evolutionary relationships and functional divergence of *CsBBELs*, homologous BBEL/BBE proteins from *C. saxicola*, closely related Papaveraceae species (*Papaver somniferum* Linnaeus, *Macleaya cordata* (Willd.) R. Br., *Corydalis yanhusuo* (Y. H. Chou and C. C. Hsu) W. T. Wang ex Z. Y. Su and C. Y. Wu, *Corydalis tomentella* Franch, and the model plant *Arabidopsis thaliana* Heynh were selected. These species were chosen to represent functionally characterized *BBELs* (*P. somniferum*, *M. cordata*), closely related congeners (*C. yanhusuo*, *C. tomentella*), and a model angiosperm for phylogenetic context (*A. thaliana*). Two phylogenetic trees were constructed using the Neighbor-Joining (NJ) method: one clustered *CsBBELs* with functionally characterized BBE/BBEL homologs (Figure 1B), and the other combined *CsBBELs* with AtBBELs (Figure 1C).

Phylogenetic analysis delineated all *BBEL* members into three distinct clades, identified as Clade I, Clade II, and Clade III (Figure 1B). The distribution of the 22 *CsBBELs* across these clades was uneven. Clade I comprised 13 *CsBBELs* and clustered with *BBEL* homologs from other Papaveraceae species, forming a species-specific lineage indicative of the primary expansion group within this family. Clade II encompassed seven members (*CsBBEL5*, *6*, *7*, *8*, *9*, *10*, *22*), which were closely associated with previously validated BIA synthesis enzymes, such as THBO1 from *C. yanhusuo* and BwSTOX from *M. cordata*, suggesting that members of this clade are primarily involved in BIA biosynthesis. Clade III contained only two members (*CsBBEL1* and *CsBBEL20*), which grouped with distant *BBEL* homologs from *P. somniferum* and *C. chinensis*.

Comparative phylogenetic analysis between *C. saxicola* and *A. thaliana* had demonstrated distinct species-specific divergence (Figure 1C). Notably, 20 out of 22 *CsBBELs* were grouped within a single independent lineage, suggesting significant species-specific expansion. In contrast, only *CsBBEL1* and *CsBBEL20* were located in other branches alongside AtBBELs. Conversely, AtBBELs were distributed across multiple clades. These findings suggested that the *BBEL* family had experienced considerable divergent evolution between *C. saxicola* and *A. thaliana*.

In conclusion, the *CsBBEL* family exhibits a high degree of conservation within the Papaveraceae, alongside notable lineage-specific expansion and functional differentiation. The close phylogenetic relationships between candidate *CsBBELs* and characterized BIA-related enzymes offer essential evolutionary insights for the identification and functional validation of core genes in subsequent research endeavors.

### 2.3. Conserved Motif and Gene Structure Analysis of CsBBELs

**Figure 2:**
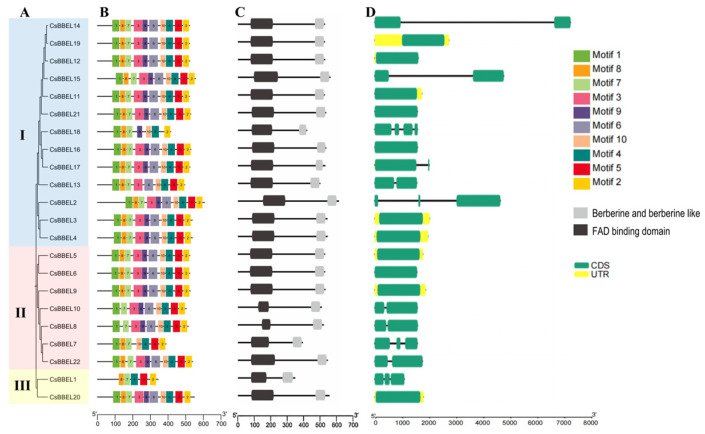
Phylogenetic relationships, conserved motifs, protein domains, and gene structures of the *CsBBELs*. (**A**) Phylogenetic tree of 22 *CsBBELs*. (**B**) Distribution of conserved motifs in *CsBBELs*, with ten distinct motifs (Motif 1–10) represented by different colored boxes. (**C**) Functional domain architecture of *CsBBELs*, where black boxes denote the FAD binding domain, and gray boxes denote the berberine and berberine-like domain. The scale bar indicates the length of the protein sequence. (**D**) Exon-intron structure of *CsBBEL* genes, with green boxes indicating coding sequences (CDS) and yellow boxes indicating untranslated regions (UTR). The scale bar indicates the length of the genomic sequence.

To elucidate the sequence conservation and structural divergence of *CsBBELs*, the conserved motifs, functional domains, and gene structures of the 22 *CsBBELs* were analyzed within the phylogenetic framework established above (Figure 2). Prior domain annotation using NCBI CDD during gene identification confirmed that all 22 *CsBBELs* possess both the BBE catalytic domain and the FAD-binding domain. The distribution of conserved motifs was highly concordant with the three-clade phylogenetic classification. Motif 2 was universally conserved across all 22 members and was localized within the BBE catalytic domain. Motif 1 and Motif 3, which mapped to the FAD-binding domain, were present in 21 and 19 members. These three motifs constitute the core functional modules of the *BBEL* family, with Motif 2 serving as the essential component of the BBE catalytic domain. Other conserved motifs exhibited distinct clade-specific distribution patterns consistent with the phylogenetic topology.

Clade I comprised 13 members with the most complete motif repertoires. In addition to the core motifs, most members of this clade harbored Motif 5 and Motif 6, with the exception of *CsBBEL18*, which lacked both, indicating a high degree of intra-clade sequence conservation. Clade II included seven members, most of which contained Motif 7 and Motif 8. *CsBBEL8* and *CsBBEL10* lacked Motif 8, and *CsBBEL7* lacked Motif 6, revealing modest sequence divergence within a conserved structural framework. Clade III consisted of only *CsBBEL1* and *CsBBEL20*, both of which displayed pronounced structural divergence at the motif level. *CsBBEL1* retained only five motifs and lacked both Motif 1 and Motif 3—the core catalytic modules of the BBE and FAD-binding domains, respectively. Although its full-length protein sequence retains identifiable BBE and FAD-binding domains based on CDD annotation, the absence of these core catalytic motifs suggests that *CsBBEL1* may be incapable of encoding a catalytically competent enzyme. This observation, together with its minimal transcript accumulation across all tested tissues, raises the possibility that *CsBBEL1* represents a pseudogenized or functionally divergent member of the family.

Analysis of gene structure further substantiated the structural divergence among clades (Figure 2D). The number of exons in *CsBBELs* ranged from one to four, with structural variations closely linked to phylogenetic grouping. Most members of Clade I were single-exon genes, accompanied by diverse multi-exon structures. Among them, *CsBBEL18* had four exons, showing obvious structural differentiation. Notably, *CsBBEL14* contained an unusually long intron (~3.5 kb), which was markedly longer than introns of other family members. Most members of Clade II underwent intron loss events, and single-exon genes dominated this clade, while a small number of genes possessed three exons. The two Clade III members exhibited divergent gene architectures: *CsBBEL1* consisted of multiple exons separated by short introns, whereas *CsBBEL20* was an intronless single-exon gene. Additionally, six *CsBBEL* genes including *CsBBEL3*, *CsBBEL4*, *CsBBEL5*, *CsBBEL9*, *CsBBEL19*, and *CsBBEL20* harbored both complete 5′ and 3′ untranslated regions. *CsBBEL12* only possessed a 5′ UTR without 3′ UTR, and *CsBBEL11* merely had a 3′ UTR. The remaining 14 genes did not carry any UTR sequences.

In summary, the conserved motif distribution, functional domain architecture, and gene organization of *CsBBELs* collectively align with the phylogenetic classification. Clade I exhibits structurally conserved features, Clade II shows moderate sequence divergence, and Clade III is characterized by pronounced structural specialization in both motif composition and gene structure. These findings provide robust sequence- and structure-level evidence to support further exploration of functional divergence among members of this family.

### 2.4. Chromosomal Localization, Duplication Events, and Collinearity Analysis of CsBBELs

**Figure 3:**
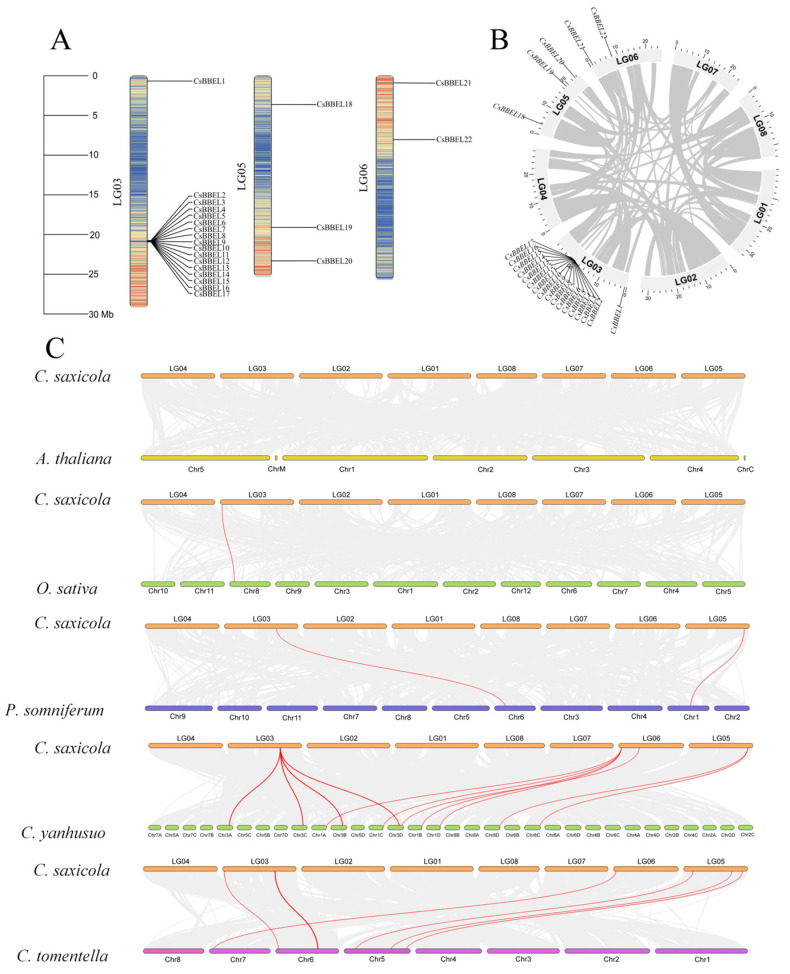
Chromosomal localization and collinearity analysis of the *CsBBELs* in *C. saxicola*. (**A**) Chromosomal localization of *CsBBEL* genes depicted on the linkage groups (LGs) of *C. saxicola*. The scale bar represents the chromosome length in megabases (Mb), with some genes exhibiting clustered distribution. (**B**) Circos plot illustrating the intra-specific collinearity of *CsBBEL* family genes in *C. saxicola*. Gray lines represent collinear gene pairs. (**C**) Inter-specific collinearity analysis of *CsBBEL* genes between *C. saxicola* and five other species, namely *A. thaliana*, *O. sativa*, *P. somniferum*, *C. yanhusuo*, and *C. tomentella*. Red lines indicate collinear orthologous gene pairs of *CsBBELs*, while the gray background signifies genome-wide collinear blocks.

To elucidate the chromosomal distribution and expansion patterns of the *CsBBELs*, a chromosomal localization map was developed utilizing the genomic annotation data of *C. saxicola* (Figure 3A; detailed information on all identified interspecific collinear gene pairs is provided in Supplementary Table S2). The *C. saxicola* genome assembly comprises 8 linkage groups (LG01–LG08), and only three of them (LG03, LG05, and LG06) harbor *CsBBEL* genes, which are accordingly the only linkage groups displayed in Figure 3A. The 22 *CsBBELs* were unevenly distributed across these three linkage groups, demonstrating a pattern that combined gene clustering with discrete arrangement. LG03 contained the largest number of members, totaling 17. Among these, *CsBBEL2*–*CsBBEL17* formed a dense cluster within the 20–25 Mb interval of LG03, whereas *CsBBEL1* was independently situated at the terminal region of the same linkage group. LG05 contained three members (*CsBBEL18*–*CsBBEL20*), and LG06 included two members (*CsBBEL21*–*CsBBEL22*), with no obvious gene clusters observed on these two linkage groups.

In conjunction with chromosomal distribution data, intraspecific collinearity and gene duplication events of *CsBBELs* were examined utilizing TBtools v2.096 (Figure 3B). No segmental duplication collinear gene pairs were identified for *CsBBELs*, suggesting that the expansion of this gene family occurred independent of whole-genome duplication or large-scale segmental duplication. According to the criteria for tandem duplication (gene interval <100 kb), the clustered *CsBBEL2*–*CsBBEL17* on LG03 were classified as tandem duplicated genes, indicating that these members originated from successive tandem duplication events. Such duplication facilitates functional divergence and neofunctionalization through the accumulation of sequence variations. The remaining members, including *CsBBEL1* and *CsBBEL18*–*CsBBEL22*, did not exhibit characteristic of tandem duplication, and their evolutionary origins warrant further investigation. Overall, tandem duplication serves as the primary driving force for the expansion of the *CsBBEL* family.

To investigate the evolutionary conservation and divergence of *CsBBELs* across different species, an interspecific collinearity analysis was conducted between *C. saxicola* and five representative angiosperms: *A. thaliana*, *O. sativa*, *P. somniferum*, *C. yanhusuo*, and *C. tomentella* (Figure 3C). The *CsBBELs* demonstrated the closest collinear relationships with closely related Corydalis species. Specifically, 11 collinear gene pairs were identified between *C. saxicola* and *C. yanhusuo*, and six pairs with *C. tomentella*. Two collinear pairs were detected with *P. somniferum* of the Papaveraceae family, only one pair with the monocot *O. sativa*, and no collinear homologs were found in the non-Papaveraceae species *A. thaliana* (detailed gene pairs are listed in Supplementary Table S2). These findings suggest that *CsBBELs* share closer genetic relatedness and higher evolutionary conservation within the genus Corydalis and have undergone distinct lineage-specific evolution during species differentiation.

### 2.5. Cis-Acting Element Analysis of *CsBBEL* Promoters

**Figure 4:**
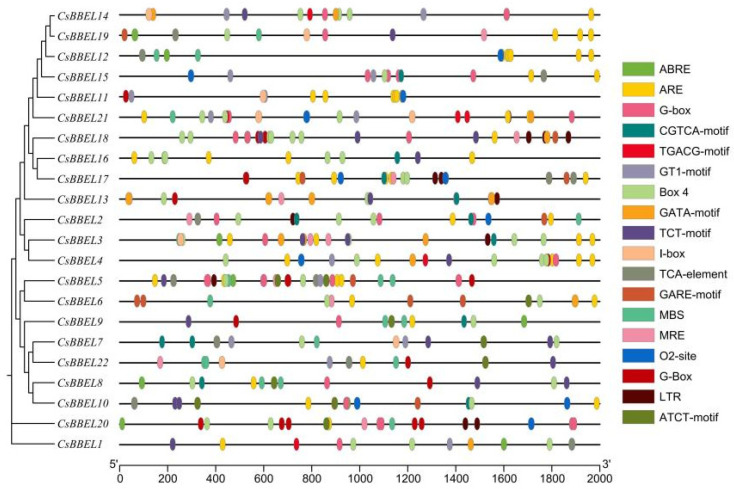
The distribution of cis-acting elements in the promoters of *CsBBELs*. The promoter region was defined as the 2.0 kb sequence located upstream of the translation initiation codon. Various types of cis-acting elements in the promoters are depicted as ellipses, each distinguished by different colors.

To examine the responses of *CsBBELs* to environmental stress and hormonal signals in *C. saxicola*, the 2.0 kb promoter sequences located upstream of the translation initiation codon (ATG) were analyzed using the PlantCARE database. Cis-acting elements related to phytohormone regulation, abiotic stress responses, and metabolic modulation were identified and characterized (Figure 4).

Three principal functional categories, encompassing five fundamental types of cis-acting elements, have been identified. (1) Abiotic stress-responsive elements include the antioxidant-responsive element (ARE), the drought-responsive MBS, light-responsive elements (G-Box, GT1-motif, Box 4), and the low-temperature-responsive LTR; (2) phytohormone-responsive elements comprise the abscisic acid-responsive ABRE, methyl jasmonate-responsive CGTCA-motif and TGACG-motif, and the salicylic acid-related TCA-element; (3) metabolic regulatory element is represented by the O2-site associated with zein metabolism. Notably, key stress and hormone elements such as ABRE, MBS, and G-Box, are extensively distributed across most *CsBBEL* promoters, suggesting that this family generally possesses the capacity to respond to environmental signals.

Several elements demonstrated distinct member-specific distribution patterns specific to individual members. The O2-site was exclusively identified in the promoters of *CsBBEL2*, *4*, *10*, *11*, *12*, *17*, *20*, and *21*. The AT-rich element, known as the ATBP-1 binding site, was observed solely in *CsBBEL17*, *18*, and *20*, whereas the light-responsive ATCT-motif was predominantly enriched in *CsBBEL10*, *18*, and *22*. This differential distribution of cis-elements offers a potential molecular basis for functional divergence, facilitating the participation of distinct *CsBBEL* members in diverse physiological processes and stress responses.

Overall, the promoters of *CsBBELs* were found to be enriched with a variety of stress- and hormone-related cis-acting elements, suggesting their significant role in environmental stress adaptation and hormone signaling. As a species endemic to karst environments, *C. saxicola* demonstrates regulation of BIA biosynthesis that is dependent on environmental and hormonal factors. Therefore, *CsBBELs* may indirectly influence BIA metabolic pathways by detecting and responding to external stress and internal hormone signals.

### 2.6. Transcriptome-Wide Expression Profiling of CsBBELs

**Figure 5:**
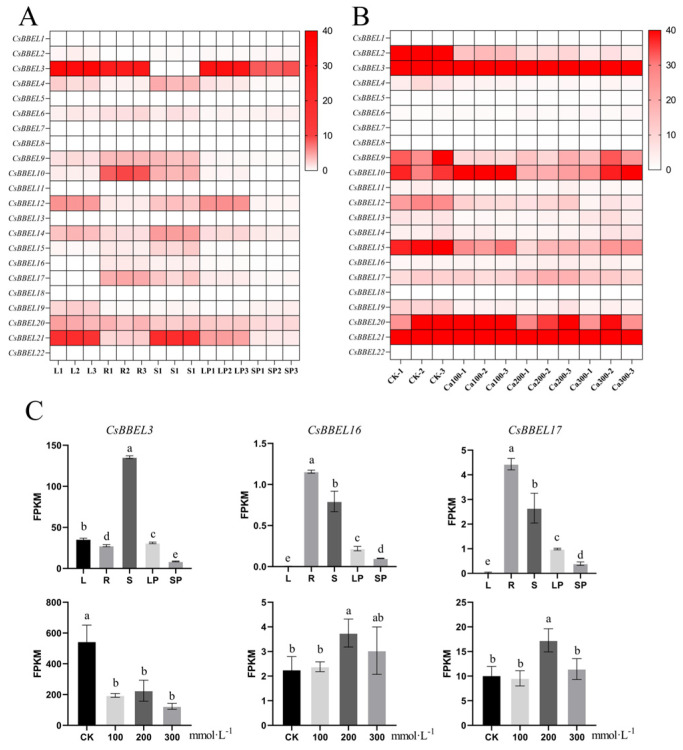
Expression patterns of *CsBBELs* in *C. saxicola*: organ-specific and calcium stress-responsive analysis. (**A**) The heatmap illustrating the organ-specific expression profiles of 22 *CsBBEL* family members across various plant organs, including leaf (L), root (R), stem (S), long petiole (LP) and short petiole (SP). The color intensity denotes the FPKM expression level, ranging from low to high (light to dark), with the *x*-axis representing biological replicates of each organ. (**B**) The heatmap depicting the expression profiles of *CsBBEL* family members under calcium gradient treatments. The color intensity indicates the FPKM expression level, ranging from low to high (light to dark), with the *x*-axis representing calcium-treated samples. (**C**) FPKM quantification of core *CsBBEL* genes presented for different organs and under calcium stress conditions. Different lowercase letters denote significant differences among groups (one-way ANOVA followed by Duncan’s multiple range test, *p* < 0.05).

Utilizing transcriptome data obtained from leaf, root, stem, long petiole, and short petiole organs of *C. saxicola*, a comprehensive analysis of the organ-specific expression patterns of *CsBBELs* was conducted, employing an expression threshold of FPKM ≥ 1 (Figure 5). The findings indicated that *CsBBEL1*, *CsBBEL5*, *CsBBEL7*, *CsBBEL8*, *CsBBEL11*, *CsBBEL13*, *CsBBEL18*, and *CsBBEL22* exhibited minimal transcript accumulation, whereas the remaining 14 family members were expressed at varying levels across different tissues. Overall, the *CsBBEL* family demonstrated distinct organ-preferential expression, with transcript abundance generally higher in roots and stems, followed by leaves. Specifically, five genes (*CsBBEL6*, *CsBBEL9*, *CsBBEL10*, *CsBBEL16*, *CsBBEL17*) were significantly upregulated in roots; another five genes (*CsBBEL3*, *CsBBEL4*, *CsBBEL14*, *CsBBEL15*, *CsBBEL21*) were highly expressed in stems; and three genes (*CsBBEL2*, *CsBBEL19*, *CsBBEL20*) exhibited predominant expression in leaves.

Utilizing transcriptome data obtained from the root tissues of *C. saxicola* subjected to exogenous calcium gradient treatments, this study further elucidated the response patterns of *CsBBEL* genes to calcium signals. A total of nine *CsBBEL* genes were identified as significantly upregulated by Ca^2+^, specifically *CsBBEL1*, *CsBBEL6*, *CsBBEL8*, *CsBBEL10*, *CsBBEL14*, *CsBBEL16*, *CsBBEL17*, *CsBBEL20*, and *CsBBEL21*. Notably, the expression of *CsBBEL6* was consistently upregulated with increasing calcium concentration. In contrast, *CsBBEL16* and the majority of other responsive genes exhibited transient upregulation or bell-shaped induction at specific calcium concentrations, indicating a clear functional divergence in calcium signal perception among members of the *CsBBEL* family.

Notably, *CsBBEL3*, *CsBBEL16*, and *CsBBEL17* demonstrated both organ-specific expression and responsiveness to calcium signals. *CsBBEL3* exhibited a high basal expression level in roots, which was generally significantly downregulated following calcium treatment, although a slight recovery was observed in the 200 mmol·L^−1^ CaCl_2_ (Ca200) group. This pattern indicates a response characterized by high basal expression and non-monotonic suppression by calcium. *CsBBEL16* was specifically expressed in roots, with minimal transcript levels detected in leaves. Its low basal expression in roots was induced in a bell-shaped manner with increasing calcium concentration, peaking in the Ca200 group, which is indicative of a typical gene response to moderate calcium stress. Similarly, *CsBBEL17* was predominantly expressed in roots and was markedly induced only under the Ca200 treatment. No significant differences in expression were observed between the low- or high-calcium groups and the control, indicating a strict specificity in its response to calcium concentration.

The observed variations in tissue expression and calcium responsiveness offer direct transcriptional evidence that substantiates the subsequent functional validation of key candidate proteins.

### 2.7. Protein Structure Prediction and Molecular Docking Analysis

Building upon prior transcriptome screening results, this study examined the binding potential of *CsBBEL3*, *CsBBEL16*, and *CsBBEL17* with benzylisoquinoline alkaloid substrates. Initially, the 3D structures of the three proteins were predicted using AlphaFold 3, with model confidence assessed through ipTM and pTM values. Structural modeling indicated that all three candidate proteins achieved an ipTM value of 0.98, with pTM values of 0.94, 0.93, and 0.91, respectively, all of which satisfied the high-confidence criteria established by AlphaFold.

**Figure 6:**
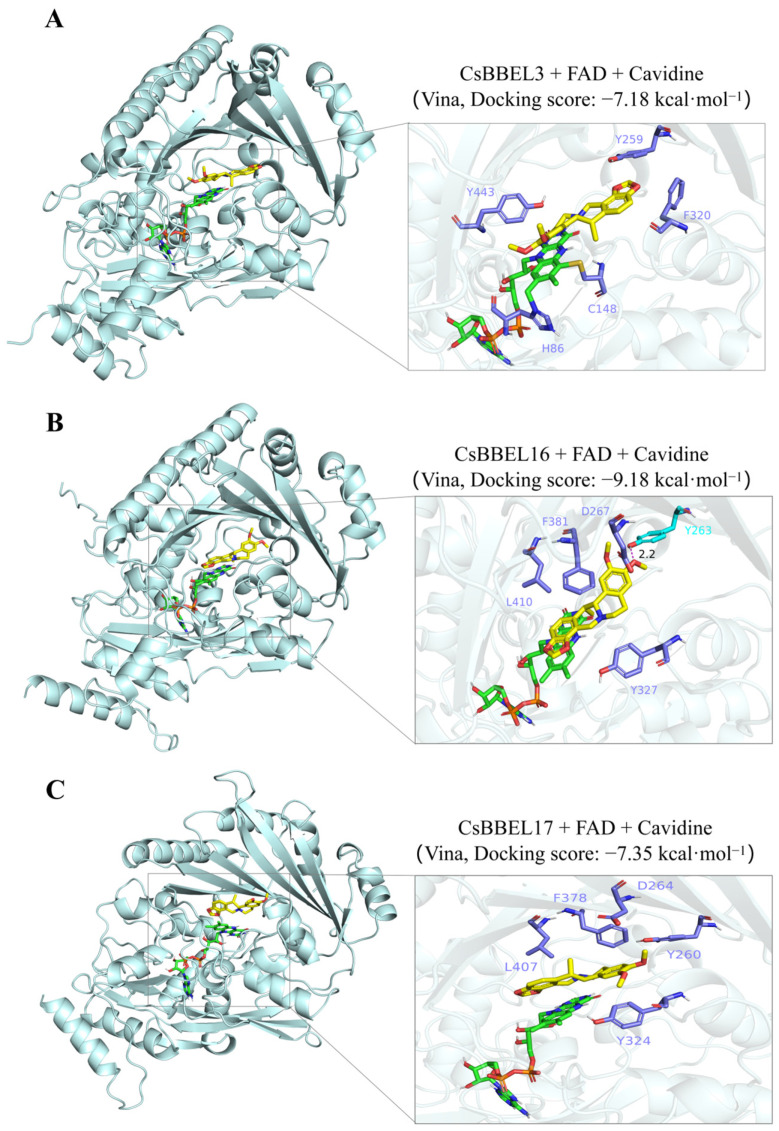
The molecular docking interactions of core *CsBBEL* proteins with FAD and cavidine. Panels (**A**), (**B**), and (**C**) depict the docking outcomes of *CsBBEL3*, *CsBBEL16*, and *CsBBEL17* with cavidine, respectively.

The holoenzyme structures complexed with the FAD cofactor were utilized as protein receptors. Two primary benzylisoquinoline alkaloids present in *C. saxicola*, namely cavidine and tetrahydrocolumbamine, were chosen as candidate ligands for molecular docking simulations conducted using AutoDock Vina v1.25. The resultant docking complexes were visualized with PyMOL v3.1. All protein–ligand combinations exhibited stable binding conformations. Upon docking with cavidine, the binding affinities of *CsBBEL3*, *CsBBEL16*, and *CsBBEL17* were determined to be −7.18, −9.18, and −7.35 kcal·mol^−1^, respectively (Figure 6A–C). For tetrahydrocolumbamine, the corresponding binding affinities were −6.23, −8.03, and −6.41 kcal·mol^−1^ (Supplementary Figure S2).

Through the integration of high-confidence structural models, reliable quality parameters, and substrate binding affinity data, *CsBBEL3*, *CsBBEL16*, and *CsBBEL17* have been identified as the principal candidate proteins responsible for BIA modification in *C. saxicola*. It is important to note that the docking scores obtained in this study were utilized solely for the relative comparison and conformational ranking of various binding modes, rather than for the quantitative prediction of absolute binding constants. Collectively, these structural analyses and docking results furnish essential structural biological support for subsequent functional experiments, including in vitro enzyme activity assays and substrate specificity verification of key candidate proteins.

### 2.8. Heterologous Expression and Activity Detection of CsBBELs

**Figure 7:**
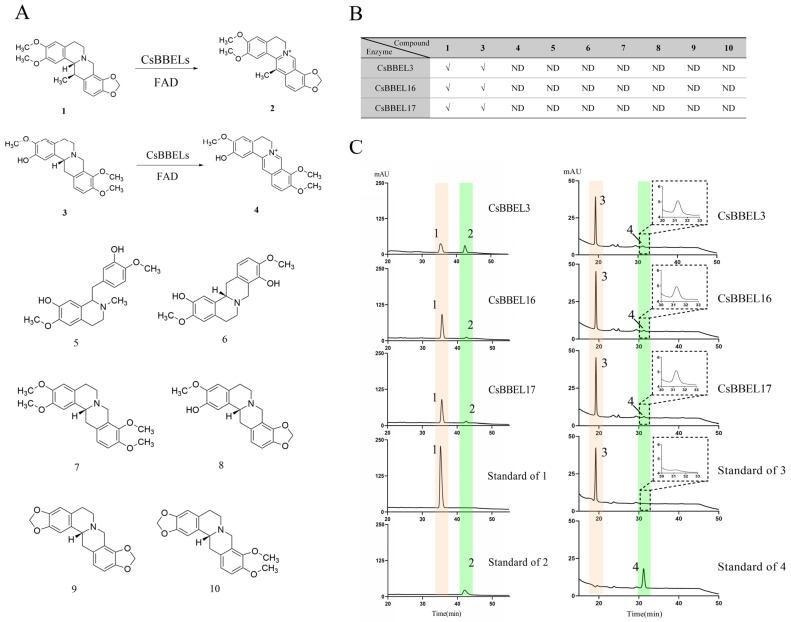
In vitro analysis of enzymatic activity and substrate specificity of core *CsBBEL* proteins from *C. saxicola*. (**A**) Chemical structures of substrates and products, alongside representative enzymatic reactions catalyzed by *CsBBEL* proteins. Typical reactions include the conversion of cavidine (1) to DHCA (2). (**B**) Results of substrate specificity screening of *CsBBEL3*, *CsBBEL16*, and *CsBBEL17*. A “√” denotes the formation of the target product, while “ND” signifies no detectable catalytic activity. (**C**) HPLC chromatograms of the enzymatic reaction products catalyzed by *CsBBEL3*, *CsBBEL16*, and *CsBBEL17*. Characteristic peaks of substrates (1/3) and products (2/4) are illustrated, with substrate and product standards included as controls to confirm product formation.

*BBEL* enzymes constitute a plant-specific subfamily within the FAD-binding oxidase superfamily, characterized by highly conserved sequences and biological functions across angiosperms. Within the BIA biosynthetic pathway, homologous BBE and *BBEL* proteins facilitate crucial two-electron and four-electron oxidation reactions, respectively. Based on these functional attributes, it is posited that the oxidation of cavidine to DHCA and tetrahydrocolumbamine to columbamine in *C. saxicola* is catalyzed by *CsBBELs* (Figure 7A).

To validate this hypothesis and systematically characterize the substrate profiles of *CsBBELs*, three core candidate proteins (*CsBBEL3*, *CsBBEL16*, and *CsBBEL17*) were heterologously expressed in Sf9 insect cells (Supplementary Figure S3). Six additional structurally diverse BIA monomers (Compounds **5**–**10**, Figure 7A) were selected as alternative substrates for in vitro enzymatic reactions. The products from all reaction systems were preliminarily screened using HPLC, and the overall activity results are summarized in Figure 7B. The results indicated that the three *CsBBELs* exhibited significant catalytic activity only toward Compound **1** (cavidine) and Compound **3** (tetrahydrocolumbamine), which were marked with “√”. No corresponding oxidized product peaks were detected for the remaining six candidate substrates (Compounds **5**–**10**), which were designated as ND (no detectable activity). These findings suggest that the three *CsBBELs* possess strict substrate specificity and exclusively recognize benzyltetrahydroisoquinoline alkaloids with specific structural skeletons.

Representative HPLC chromatograms of the two positive reaction systems are presented to elucidate the chromatographic characteristics indicative of effective enzymatic catalysis (Figure 7C). Product peaks corresponding to the retention time of the DHCA standard (Compound **2**) were identified in reactions supplemented with Compound **1**. Similarly, product peaks consistent with the columbamine standard (Compound **4**) were detected in the reaction system containing Compound **3**. No target product signals were observed in the parallel enzyme-free negative control groups. Subsequent liquid chromatography–mass spectrometry (LC-MS) analysis further confirmed that the molecular weights and characteristic fragment ions of the catalytic products were fully consistent with the theoretical mass spectral data of DHCA (Compound **2**) and columbamine (Compound **4**) (Supplementary Figure S4). These results provide compelling evidence for the catalytic specificity of *CsBBELs* and the structural accuracy of their reaction products.

## 3. Discussion

In this study, we conducted the inaugural genome-wide identification of 22 *CsBBELs* in *C. saxicola*, each possessing the conserved FAD-binding domain and BBE catalytic domain characteristic of the *BBEL* family. Phylogenetic analysis categorized these members into three distinct clades. The *CsBBEL2–17* tandem duplication cluster on linkage group 03 (LG03) facilitates the expansion of the *CsBBEL* family, with notable structural and functional divergence observed among different clades. Analyses of cis-acting elements and expression profiles revealed that *CsBBELs* are predominantly expressed in roots and stems. Notably, *CsBBEL3*, *CsBBEL16*, and *CsBBEL17* exhibit root-specific expression patterns, alkaloid oxidase catalytic activity, and significant Ca^2+^ responsiveness. Building on these findings, we discuss the molecular strategy underlying DHCA-specific accumulation in *C. saxicola* in karst habitats, focusing on two dimensions: differential expression regulation of isozymes and catalytic substrate specialization.

### 3.1. Differential and Coordinated Regulation of Isozymes: A Molecular Strategy for C. saxicola to Adapt to Calcium Fluctuations in Karst Habitats

Karst regions are distinguished by significant spatiotemporal variability in soil calcium concentrations, with levels fluctuating by as much as 3–41-fold across different microhabitats [13]. In response to these substantial variations in calcium availability, *C. saxicola* requires both sustain basal homeostatic synthesis of DHCA and rapid upregulation of production under high Ca^2+^ stress. Our study identified that the core isozymes *CsBBEL3*, *CsBBEL16*, and *CsBBEL17* demonstrate considerable functional redundancy in their catalytic activities, each capable of facilitating the four-electron oxidation of cavidine and tetrahydrocolumbamine (Figure 7). Nonetheless, their Ca^2+^ response patterns exhibit significant divergence: *CsBBEL3* maintains constitutively stable expression and remains unresponsive to exogenous Ca^2+^, whereas *CsBBEL16* and *CsBBEL17* are markedly upregulated following exposure to 200 mmol·L^−1^ CaCl_2_ (Figure 5).

This combination of functional redundancy and differential regulation appears to represent a key adaptive strategy for *C. saxicola* to cope with Ca^2+^ fluctuations in karst habitats. *CsBBEL3*, serving as a basal homeostatic maintenance isozyme, facilitates the continuous synthesis of DHCA across diverse Ca^2+^ conditions. Conversely, *CsBBEL16* and *CsBBEL17* act as Ca^2+^-inducible isozymes that are swiftly activated under high-Ca^2+^ stress, which can boost biosynthetic flux to alleviate oxidative damage and biotic stress. Such regulatory patterns are widely observed in plant secondary metabolism. For instance, isozymes of anthranilate synthase in *A. thaliana* coordinately regulate indole secondary metabolism through differential stress responses; UDP-glycosyltransferase isozymes in *Platycodon grandiflorus* maintain basal saponin synthesis via constitutive high expression under normal conditions, while specific isozymes are strongly induced under drought stress [14]; the cytochrome P450 isozyme family in *Chrysanthemum indicum* similarly exhibits a clear division between constitutive and inducible members [15]. Unlike these previous cases, the functional division of *CsBBELs* in *C. saxicola* is closely linked to Ca^2+^—the defining environmental factor of karst habitats. This observation is consistent with adaptive matching between secondary metabolic regulation and specific ecological factors.

Notably, the three core isozyme genes are all situated within the *CsBBEL2–17* tandem duplication cluster on linkage group 03 (LG03) (Figure 3A). This genomic configuration provides a valuable clue for exploring the origin of their regulatory divergence. The immediate outcome of tandem duplication is the creation of multiple gene copies with high sequence similarity; its widely recognized evolutionary role is to provide genetic material for subsequent functional divergence [15]. In alignment with this evolutionary framework, Shan et al. [16] discovered that key enzymes involved in benzylisoquinoline alkaloid (BIA) biosynthesis in Houttuynia cordata, such as 6OMT and CYP80B, form conserved gene clusters through tandem duplication, with homologous genes undergoing functional divergence and subfunctionalization. Similarly, Peng et al. [17] confirmed that homologous genes in the O-methyltransferase tandem cluster of Citrus species coordinately regulate polymethoxyflavone biosynthesis through divergent expression patterns. In the single-copy state, genes are generally subject to stringent purifying selection constraints, and mutations that impair ancestral function are infrequently retained. Conversely, redundant copies generated through tandem duplication alleviate these constraints: one copy may preserve the ancestral functional role, while the other acquires a relatively unrestricted mutational space [18]. These mutations, when they stochastically confer Ca^2+^-responsive expression traits on a gene, may be favored by natural selection as they enable plants to enhance the biosynthetic rate of DHCA under high-Ca^2+^ stress. *CsBBEL3* likely represents the copy that retains the ancestral expression pattern, thereby maintaining basal biosynthetic homeostasis. In contrast, *CsBBEL16* and *CsBBEL17* probably acquired novel calcium-inducible expression traits through gradual regulatory mutations following duplication. This functional division of “basal homeostatic maintenance + precise stress regulation”, initiated by tandem duplication and refined by natural selection, provides a critical genetic basis for *C. saxicola* to adapt to the drastic fluctuations in Ca^2+^ concentrations in karst habitats.

Interspecific collinearity analysis has demonstrated that no collinear orthologous segments of *CsBBELs* are present in *A. thaliana*, whereas there are extensive conserved collinear relationships within the Papaveraceae family. This observation suggests that the *BBEL* family has experienced lineage-specific expansion exclusively within the Papaveraceae. Catalytic enzyme genes from Corydalis and other genera within Papaveraceae maintain orthologous relationships solely within the family, while gene loss has occurred in more distantly related outgroup species. At the metabolic level, *A. thaliana* lacks the genetic capacity to synthesize endogenous BIAs, consistent with the well-documented independent loss of core BIA biosynthetic genes in nearly all core eudicot lineages [19]. In contrast, functional BIA biosynthetic capacity has been consistently verified in all experimentally characterized Papaveraceae genera, including *Corydalis*, *Papaver*, *Macleaya*, *Glaucium*, and *Argemone* [20]. This distribution pattern of *BBEL* genes is highly consistent with their biological role in BIA biosynthesis. It is widely recognized that the selective advantage conferred by alkaloid biosynthesis has driven the diversification of the *BBEL* family within the Papaveraceae, whereas Brassicaceae species such as *A. thaliana* have not retained functional orthologs of these key biosynthetic genes.

### 3.2. Substrate Narrowing of Homologous Enzymes: The Catalytic Mechanism Underlying the Specific Accumulation of Dehydrocavidine in C. saxicola

In addition to the divergence observed at the level of expression regulation, *CsBBEL3*, *CsBBEL16*, and *CsBBEL17* demonstrate significant functional specialization at the catalytic function level. In vitro enzymatic activity assays have revealed that these three isozymes exclusively catalyze the oxidation of cavidine and tetrahydrocolumbamine, while exhibiting no detectable catalytic activity toward the other six structurally related alkaloid precursors (Figure 7B,C). This narrowing of substrate selectivity poses a fundamental evolutionary question: how did this specialization evolve stepwise from a presumably more promiscuous ancestral enzyme? Tandem duplication may provide the essential genetic prerequisite for this process, while sequence variations in substrate-binding regions and selective pressures imposed by karst environments may collectively shape the direction of this specialization.

From an evolutionary perspective of the *BBEL* gene family, Clade I and Clade II (which encompasses promiscuous catalytic enzymes such as THBO from *C. chinensis* [8]) represent two principal pathways of functional divergence of *BBEL* proteins within Ranunculales. Clade II is thought to retain the ancestral promiscuous catalytic capacity and is involved in multiple BIA biosynthetic pathways. Conversely, Clade I may have experienced an evolutionary shift toward substrate specialization in the common ancestor of the genus Corydalis, after which various species further diverged to develop their own preferred substrate types within this framework. Notably, homologous *BBEL* enzymes in Clade I from *C. tomentella* also demonstrate substrate specialization, yet they recognize distinct substrate types compared to *C. saxicola* [9,11]. This suggests that functional divergence following tandem duplication occurs not only at the regulatory level but also at the catalytic selectivity level—homologous enzymes from the two species have independently diverged in substrate preference while sharing the same evolutionary trajectory toward substrate specialization.

From a structural biology perspective, the substrate selectivity of *BBEL* oxidases is predominantly determined by the amino acid residues located within their substrate-binding regions [21,22]. Notably, *BBEL* homologous genes within the genus Corydalis are subjected to global purifying selection (Ka/Ks < 1) [23], suggesting that their core catalytic structures are highly conserved. Functional divergence is likely not achieved through large-scale sequence remodeling but is instead driven by a limited number of amino acid substitutions within the substrate-binding regions. Tandem duplication may provide the genetic space necessary for such precise functional tuning: redundant copies are liberated from the stringent selective pressures imposed on single-copy genes, thereby allowing their substrate-binding regions to accommodate a certain degree of sequence variation. When a substitution serendipitously enhances the binding affinity or catalytic efficiency toward a specific substrate (e.g., cavidine), this variant may be favored by natural selection.

Within the specific selective context of karst habitats, the evolutionary rationale for this substrate narrowing is largely coherent. DHCA serves as the principle bioactive component of *C. saxicola*, conferring resistance to high-calcium stress, drought stress, and biotic challenges in karst environments. We propose that by concentrating the catalytic capacity of rate-limiting enzymes on one or two key substrates, *C. saxicola* effectively channels limited carbon skeletons and energy into the DHCA biosynthetic pathway, thereby preventing resource wastage due to the unregulated diversion of metabolic flux into multiple secondary metabolites. In this regard, the narrowing of substrate selectivity in *BBEL* enzymes does not signify a reduction in catalytic function but may rather represent a metabolic precision strategy evolved by plants during long-term adaptation to specific habitats. Such substrate specialization driven by lineage-specific evolution of biosynthetic enzymes is a widely observed pattern in BIA biosynthetic pathways [24,25]. It may channel broad metabolic potential into precise ecological functions, ultimately facilitating the specific accumulation of DHCA in *C. saxicola*.

In summary, the evolution of the *CsBBEL2–17* tandem duplication cluster may facilitate the two-dimensional coordinated divergence of isozymes. Firstly, at the level of expression regulation, a functional division characterized by “basal homeostatic maintenance + precise stress regulation” is established. Secondly, at the catalytic function level, substrate selectivity is narrowed. These two facets collectively form the core molecular strategy proposed of *C. saxicola* for adaptation to karst environments. Divergence in expression regulation imparts a dynamic balance capacity to DHCA synthesis in response to fluctuations in calcium concentration, while substrate narrowing allows for the precise allocation of metabolic resources toward DHCA biosynthesis.

This study systematically characterizes the evolutionary traits and catalytic functions of the *CsBBEL* family in *C. saxicola*, yet several significant limitations persist. The catalytic activities of only three core members have been validated in vitro, and there is a lack of genetic evidence regarding their in vivo physiological functions and coordinated regulatory patterns. The role of calcium-responsive elements in the promoter regions of tandem-duplicated genes and the upstream regulatory pathways governing calcium-induced expression remain uncharacterized. Additionally, the direct causal relationship between *CsBBEL* substrate specificity and the specific accumulation of DHCA has not been established. Future research should aim to elucidate the relevant molecular mechanisms through experiments such as gene silencing and overexpression, promoter functional validation, dissection of calcium signaling networks, and systematic enzymatic activity characterization of the entire *CsBBEL* family. These efforts will provide theoretical support for the germplasm conservation and molecular breeding of *C. saxicola*.

## 4. Materials and Methods

### 4.1. Plant Materials and Treatments

The experimental specimens of *C. saxicola* Bunting were sourced from Donglan County, Hechi City, Guangxi Zhuang Autonomous Region, and subsequently cultivated and maintained at the Guangxi Botanical Garden of Medicinal Plants. A voucher specimen (No. 0317019) has been deposited in the Guangxi Herbarium of Traditional Chinese Medicine. The collection of wild specimens was conducted under the authorization of a collection permit for national Class II protected wild plants (Permit No. Guizhicaizheng 0030784).

Seeds were soaked overnight in clean water, sown in a sand:vermiculite = 1:1 (*v*/*v*) seedling substrate, and cultured in an artificial climate chamber at 25 °C, 60% relative humidity, 100 μmol·m^−2^·s^−1^ light intensity with a 12 h light/12 h dark photoperiod. For organ-specific expression analysis, 30-day-old uniform seedlings were transplanted into individual 25 cm (d) × 20 cm (h) plastic pots (one seedling per pot) under the same environmental conditions as previously described, with uniform watering. After 150 days of further cultivation, fresh and healthy tissues including leaves, roots, stems, long petioles, and short petioles were harvested between 9:00 and 10:00 a.m. For calcium stress treatment, an independent batch of one-month-old seedlings was grown in pots containing a peat soil:vermiculite matrix (3:1, *v*/*v*) in a greenhouse at 23 °C, with a 16 h photoperiod and a light intensity of 120 μmol·m^−2^·s^−1^. Seedlings were irrigated with equal volumes of CaCl_2_ solution at concentrations of 0, 100, 200, and 300 mmol·L^−1^ for 25 consecutive days. Root tissues were harvested separately. All samples were immediately frozen in liquid nitrogen and stored at −80 °C.

### 4.2. Genome-Wide Identification, Physicochemical Properties and Phylogenetic Analysis of CsBBELs

The hidden Markov model (HMM) profiles of the BBE domain (PF08031) and FAD-binding domain (PF01565) were retrieved from the Pfam database, and homologous searches against the genome-wide protein sequences of *C. saxicola* were performed using HMMER v3.4 software with an E-value cutoff of 1e^−5^ to obtain preliminary candidate sequences of *CsBBELs* [26]. Subsequently, 28 *Arabidopsis* *BBEL* proteins (AtBBELs) retrieved from The Arabidopsis Information Resource (TAIR) database were employed as query sequences. A secondary BLASTp homology screening was performed using the built-in BLAST v2.2.25 tool in TBtools v2.096 [27], with screening thresholds set at an E-value ≤ 1 × 10^−5^, sequence identity ≥40%, and alignment coverage ≥50%. The integrity of characteristic conserved domains in the retained sequences was further validated through the InterPro [28] and SMART databases [29]. Following the removal of redundant sequences, the final members of the *BBEL* gene family in *C. saxicola* were determined.

The ExPASy online server (https://web.expasy.org/protparam/; accessed on 24 September 2025) [30] was employed to examine the physicochemical properties of *CsBBEL*, including amino acid length, molecular weight, isoelectric point, hydropathicity, and instability index. Additionally, the online tools Wolf-psort and Cell-PLoc were utilized to predict the subcellular localization of candidate proteins.

Additionally, to enhance the reliability of subcellular localization prediction, five independent online tools were utilized for candidate proteins, namely WoLF PSORT (https://wolfpsort.hgc.jp/; accessed on 24 September 2025) [31], Cell-PLoc 2.0 (http://www.csbio.sjtu.edu.cn/bioinf/Cell-PLoc-2/; accessed on 24 September 2025) [32], CELLO (https://cello.life.nctu.edu.tw/; accessed on 24 September 2025), PSORT2 (https://www.genscript.com/psort.html) [33] and Euk-mPLoc 2.0 (http://www.csbio.sjtu.edu.cn/bioinf/euk-multi-2/; accessed on 24 September 2025) [34]. For each *CsBBEL* protein, the number of tools supporting each subcellular compartment was counted to generate a consensus vote matrix. The matrix was further subjected to row-wise Z-score standardization, which normalizes the vote counts against the mean and standard deviation of each individual protein across all compartments, to highlight the relative localization preference of each family member. After standardization, the values ranged approximately from −2.50 to 2.50, with positive values indicating stronger localization preference than the average level of the corresponding protein, and negative values indicating weaker preference. The standardized values were used for subsequent visualization.

BBEL protein sequences were retrieved from *C. saxicola* and four BIA-producing species (*P. somniferum* [10], *M. cordata* [9], *C. chinensis* [8], *Eschscholzia californica Cham.* [5]). All phylogenetic trees were constructed via the Neighbor-Joining (NJ) method in MEGA 11.0 with 1000 bootstrap replicates, using the Poisson model for amino acid substitution and pairwise deletion for gap-containing loci [35]. Two distinct phylogenetic trees were generated for different analytical purposes. One clustered *CsBBELs* with homologs from the four aforementioned BIA-producing species. The other compared *CsBBELs* with AtBBELs to further resolve the evolutionary relationships of the *CsBBEL* family. All trees were visualized and optimized using the online tool iTOL (https://itol.embl.de/) [36]. Sequence accessions of all *CsBBEL* genes used in the phylogenetic analyses are summarized in Supplementary Table S1.

### 4.3. Chromosomal Localization, Gene Duplication, and Collinearity Analysis

The chromosomal locations of the *CsBBELs* were extracted from the GFF3 annotation file of the *C. saxicola* genome, and their chromosomal distribution map was generated using TBtools v2.096 [37]. Genomic data for interspecific collinearity analyses were obtained from public databases: the genomes of *A. thaliana* and *O. sativa* were retrieved from NCBI (https://www.ncbi.nlm.nih.gov/datasets/genome/GCF_000001735.4/; accessed on 1 July 2025; https://www.ncbi.nlm.nih.gov/datasets/genome/GCF_034140825.1/; accessed on 1 July 2025); *P. somniferum* and *C. tomentella* were obtained from the China National GenBank Sequence Archive (https://ngdc.cncb.ac.cn/gwh/Assembly/139/show; accessed on 1 July 2025; https://ngdc.cncb.ac.cn/gwh/Assembly/10358/show; accessed on 1 July 2025); *C. yanhusuo* were obtained from CNGBdb (https://db.cngb.org/data_resources/project/CNP0004356; accessed on 1 July 2025). The high-quality de novo genome of *C. saxicola* was assembled by BGI Genomics (Shenzhen, China).

TBtools v2.096 was utilized for the analysis of intraspecific collinearity in *C. saxicola* and for interspecific collinearity comparisons with the aforementioned five species, with parameters configured to a minimum match score of 50 and a maximum gap of 20 [37]. Tandem duplications were characterized as two or more contiguous homologous genes located on the same chromosome, whereas whole-genome duplication (WGD) events were identified based on homologous pairs located on different chromosomes [9]. The classification of gene duplication, collinearity analysis, and visualization were all conducted using TBtools v2.096 [37].

### 4.4. Conserved Motif, Gene Structure, and Cis-Acting Element Analysis

The conserved motifs of *CsBBELs* were examined utilizing MEME Suite 5.5.7 [38], with the maximum number of conserved motifs designated as ten. Utilizing the genome GFF3 annotation and CDS of *C. saxicola*, the exon–intron structure of *CsBBELs* was visualized through TBtools v2.096 [37]. The 2 kb upstream sequences from the translation start site of *CsBBELs* were extracted, and cis-acting elements were predicted and annotated using the PlantCARE database [27].

### 4.5. Transcriptome Sequencing and Expression Pattern Analysis

Total RNA was extracted from various organs and Ca^2+^-treated root samples of *C. saxicola* using the MolPure^®^ Plant Total RNA Kit (Yeasen Biotechnology, Shanghai, China). The integrity, concentration, and purity of the RNA were evaluated using 1% agarose gel electrophoresis and UV-Vis spectrophotometer (NanoDrop 2000, Thermo Fisher Scientific, Waltham, MA, USA). Three independent biological replicates were set for each organ type and each calcium treatment group. RNA samples that met the quality criteria were utilized to construct transcriptome libraries in accordance with standard Illumina protocols and subsequently sequenced on the Illumina NovaSeq 6000 platform (Illumina, San Diego, CA, USA). Raw sequencing data were subjected to quality control and filtering using SOAPnuke v1.5.6 [39]. Following adapter trimming and the elimination of low-quality reads with Fastp, the clean reads were aligned to the *C. saxicola* reference genome utilizing HISAT2 v2.2.1 [40]. Gene expression levels were quantified at the transcript level using Stringtie v2.1.4 and normalized to FPKM values [41]. The organ-specific and Ca^2+^-responsive expression patterns of *CsBBELs* were analyzed and visualized using TBtools v2.096 [37].

Statistical analysis of gene expression quantification data was performed with IBM SPSS Statistics version 22. One-way analysis of variance (ANOVA) was first conducted to detect overall significant differences among organ groups and calcium gradient treatment groups, and Duncan’s multiple range test was subsequently applied for pairwise multiple comparisons of means, with a significance threshold set at *p* < 0.05. All quantitative plots were generated using GraphPad Prism 8 software.

### 4.6. Protein Structure Prediction and Molecular Docking

The three-dimensional structural modeling of the core candidate *CsBBELs* was executed utilizing AlphaFold3, with model confidence assessed through ipTM and pTM values, ipTM (interface predicted TM-score) > 0.8 and pTM (Predicted Template Modeling) > 0.5 were considered as high-confidence predictions [42]. The stereochemical qualities of the final representative models were further validated via PROCHECK Ramachandran plot analysis (https://saves.mbi.ucla.edu/; accessed on 15 July 2026) (Supplementary Figure S1) [43]. Representative BIA compounds, such as cavidine, tetrahydropalmatine, and cheilanthifoline, were chosen as potential ligands for molecular docking analyses conducted with AutoDock Vina [44]. Prior to docking, redundant water molecules were excised from the protein receptors, followed by the addition of hydrogen atoms and optimization of charges. All ligand structures underwent further refinement through energy minimization. Subsequent to the docking simulations, the binding affinities and key interaction sites between the proteins and ligands were summarized. PyMOL was employed to visualize the three-dimensional structures of the proteins, the binding modes of the ligands, and the resulting docking complexes [45].

### 4.7. Gene Cloning and Heterologous Expression Vector Construction

Total RNA was extracted from the root tissues of *C. saxicola*, and genomic DNA contamination was eliminated through DNase I digestion. First-strand cDNA was synthesized via reverse transcription and subsequently utilized as the template for PCR amplification. Specific primers were designed based on the coding sequence (CDS) regions of the candidate genes *CsBBEL3*, *CsBBEL16*, and *CsBBEL17* (Supplementary Table S3). Full-length CDS fragments were amplified by PCR. The amplicons were ligated into a cloning vector and transformed into *E. coli*; for each target gene, 8 positive colonies were preliminarily screened by PCR, and 4 clones with expected band sizes were selected for Sanger sequencing to verify sequence accuracy. Following sequence verification, the amplified fragments were codon-optimized and cloned into the pFastBacDual vector to generate recombinant heterologous expression constructs for subsequent production of recombinant proteins [46]. Noteworthily, only synonymous nucleotide substitutions were introduced during codon optimization, and the encoded amino acid sequences were completely identical to those of native *CsBBEL3*, *CsBBEL16*, and *CsBBEL17*.

### 4.8. Heterologous Expression and Purification of Recombinant Proteins

The Bac-to-Bac baculovirus expression system was utilized for the production of heterologous proteins [47]. Verified recombinant plasmids were transformed into *Escherichia coli* DH10Bac competent cells (Thermo Fisher Scientific, Waltham, MA, USA), and positive colonies were selected to isolate recombinant bacmid DNA. The purified bacmid was subsequently transfected into Sf9 insect cells at a density of 2 × 10^6^ cells·mL^−1^. The primary P1 recombinant baculovirus was harvested 48–72 h post-transfection and was further amplified for two to three rounds to produce high-titer P3 viral stocks.

During the logarithmic growth phase, Sf9 cells were infected with P3 virus at a multiplicity of infection (MOI) of 1:200 and cultured in suspension at 27 °C with agitation at 180 rpm for 48–72 h to drive the expression of target *CsBBEL* genes and produce the corresponding recombinant proteins. Post-cultivation, cells were harvested, lysed via ultrasonication, and centrifuged to obtain the supernatant. His-tagged recombinant proteins were subsequently purified through Ni affinity chromatography, with impurity proteins being eliminated via gradient imidazole elution, and fractions containing the target protein were collected. The purity of the proteins was evaluated using SDS-PAGE, and protein concentration was quantified with the BCA Protein Assay Kit (Thermo Fisher Scientific, Waltham, MA, USA). The purified proteins were concentrated by ultrafiltration, aliquoted, and stored at −80 °C for subsequent in vitro enzymatic activity assays.

### 4.9. In Vitro Enzymatic Reaction and Product Detection

An in vitro enzymatic reaction and product detection system was developed based on previously reported alkaloid analytical methods for *C. tomentella* [11]. Eight alkaloid substrates were utilized, including cavidine, cheilanthifoline, tetrahydrocolumbamine, tetrahydropalmatine, reticuline, scoulerine, stylopine, and tetrahydroberberine. A reaction mixture of 100 μL was prepared, comprising 100 mmol·L^−1^ Tris-HCl buffer (pH 8.89), 100 μmol·L^−1^ substrate, and 1 μg of purified recombinant *CsBBEL*. Following incubation at 27 °C for 6 h, the reaction was terminated by the addition of 50 μL of chromatographic-grade methanol. The mixture was then centrifuged at 12,000 rpm for 10 min, and the supernatant was collected for subsequent analysis. All eight substrate reactions were set up in parallel using the same batch of purified recombinant protein, incubated simultaneously, and processed for detection under identical conditions. Each *CsBBEL*–substrate combination was performed in three independent biological replicates; all replicates used the same batch of purified recombinant protein, with independently prepared reaction mixtures and separate incubation and detection procedures.

Preliminary product screening was conducted utilizing an Agilent 1260 high-performance liquid chromatograph (HPLC) (Agilent Technologies, Santa Clara, CA, USA) equipped with an Xbridge BEH C18 column (4.6 mm × 250 mm, 5 μm). The mobile phase comprised acetonitrile (A) and an aqueous solution containing 0.2% phosphoric acid, 20 mmol·L^−1^ potassium dihydrogen phosphate, and 10 mmol·L^−1^ triethylamine (B). The gradient elution program was structured as follows: 15–22% A (0–10 min), 22% A maintained constant (10–40 min), and 22–15% A (40–50 min). The flow rate was set at 0.8 mL·min^−1^, the detection wavelength was 280 nm, the column temperature was maintained at 25 °C, and the injection volume was 10 μL. Preliminary identification of substances was achieved by comparing retention times with those of authentic standards. A reaction was classified as catalytically active (marked as “√”) when the target product peak matching the retention time of the authentic standard was detected; reactions with no detectable target product peak were designated as “ND” (no detectable activity). Potential non-specific degradation of substrates into uncharacterized byproducts was not considered in this activity classification.

Samples that yielded positive results via HPLC underwent further analysis utilizing an Agilent 1290 ultra-high-performance liquid chromatography system, which was coupled with a 6550A quadrupole time-of-flight mass spectrometer (UPLC-Q-TOF-MS). The separation process was conducted on an Agilent Zorbax SB C18 column (100 mm × 2.1 mm, 3.5 μm). The mobile phase comprised 0.1% formic acid in water (A) and 0.1% formic acid in methanol (B). The gradient program was established as follows: 10–20% B (0–3 min), 20–25% B (3–13 min), 25–30% B (13–23 min), and 30–40% B (23–33 min). The flow rate was maintained at 0.3 mL·min-1, the column temperature was set at 35 °C, and the injection volume was 5 μL.

Mass spectrometry was conducted in positive ion mode, employing automatic MS/MS scanning over an m/z range of 50–500. The MS parameters were set as follows: drying gas temperature at 200 °C, drying gas flow rate at 16 L·min^−1^, nebulizer pressure at 344.7 kPa, sheath gas temperature at 350 °C, sheath gas flow rate at 12 L·min^−1^, capillary voltage at 3500 V, nozzle voltage at 500 V, and collision energy at 20 eV. The catalytic products were subsequently characterized using Agilent MassHunter Profinder software B.06.00, utilizing the fragment ion information derived from standard compounds. Positive reaction combinations were selected for mass spectrometric validation, and each sample was analyzed in three technical replicates to confirm the molecular weight and characteristic fragment ions of the catalytic products.

## 5. Conclusions

This study represents the inaugural genome-wide identification of the *CsBBEL* gene family in *Corydalis saxicola* Bunting, identifying a total of 22 members, each containing the conserved FAD-binding domain and BBE catalytic domain. In vitro functional validation demonstrated that *CsBBEL3*, *CsBBEL16*, and *CsBBEL17* specifically catalyze the four-electron oxidation of cavidine and tetrahydrocolumbamine, functioning as key rate-limiting enzymes in DHCA biosynthesis. Our findings reveal that tandem duplication-driven divergence in expression regulation and catalytic functional specialization constitutes the core molecular mechanism by which *C. saxicola* adapts to high-calcium karst habitats and specifically accumulates DHCA. This study establishes a foundation for elucidating the environmental regulatory mechanisms of secondary metabolism in *C. saxicola* and provides an important theoretical basis for its germplasm conservation and medicinal quality improvement.

## Figures and Tables

**Table 1:** Identification and physicochemical analysis of *CsBBELs*.

Sequence ID	Number of Amino Acid	Molecular Weight	Theoretical pI	Instability Index	Aliphatic Index	Grand Average of Hydropathicity
*CsBBEL1*	343	38,716.17	5.74	39.2	89.45	−0.067
*CsBBEL2*	606	68,410.12	8.05	33.48	85.71	−0.225
*CsBBEL3*	537	60,594.52	9.17	34.06	82.96	−0.246
*CsBBEL4*	538	60,967.07	9.44	33.58	84.22	−0.261
*CsBBEL5*	523	58,906.72	9.1	44	92.96	−0.166
*CsBBEL6*	523	58,901.22	7.1	38.31	91.84	−0.217
*CsBBEL7*	389	43,866.54	9.58	30.92	83.91	−0.193
*CsBBEL8*	515	57,981.51	9.48	40.62	86.27	−0.23
*CsBBEL9*	525	58,730.15	8.55	36.74	88.78	−0.173
*CsBBEL10*	503	56,425.45	9.17	41.05	85.81	−0.141
*CsBBEL11*	521	58,182.5	7.65	36.75	88.12	−0.112
*CsBBEL12*	523	58,771.91	6.81	33.76	85.72	−0.169
*CsBBEL13*	494	55,431.37	8.4	43.2	87.83	−0.046
*CsBBEL14*	522	58,625.11	8.83	33.93	87.74	−0.123
*CsBBEL15*	555	61,828.35	9.44	29.69	88.54	−0.066
*CsBBEL16*	530	59,925.84	8.61	34.62	91.34	−0.145
*CsBBEL17*	524	59,189.17	9.04	34.53	91.09	−0.155
*CsBBEL18*	415	46,981.87	9.08	32.22	83.78	−0.221
*CsBBEL19*	522	58,507.82	8.59	32.78	87.72	−0.171
*CsBBEL20*	549	61,007.41	5.21	33.38	90.77	−0.13
*CsBBEL21*	529	59,446.99	8.97	36.44	89.15	−0.197
*CsBBEL22*	539	60,885.9	8.95	34.37	88.05	−0.188

## Data Availability

The original contributions presented in this study are included in the article/Supplementary Materials. Further inquiries can be directed to the corresponding authors.

## Supplementary Materials

The following supporting information can be downloaded at: https://www.mdpi.com/article/10.3390/ijms27156708/s1.

## Abbreviations

The following abbreviations are used in this manuscript:

BIAs	Benzylisoquinoline alkaloids
BBEL	Berberine Bridge Enzyme-Like
DHCA	Dehydrocavidine
DBOX	Dihydrobenzophenanthridine oxidases
CDS	Coding Sequence
MOI	Multiplicity of Infection
HMM	Hidden Markov Model
FAD	Flavin Adenine Dinucleotide
NJ	Neighbor-Joining
FPKM	Fragments Per Kilobase of transcript per Million mapped reads
HPLC	High Performance Liquid Chromatography
SDS-PAGE	Sodium Dodecyl Sulfate Polyacrylamide Gel Electrophoresis
Ka/Ks	Non-synonymous substitution rate/Synonymous substitution rate
VAO/PCMH	Vanillyl-alcohol oxidase/p-cresol methylhydroxylase superfamily
STOX	Stylopine oxidase
6OMT	6-O-methyltransferase
