# Supplementary material for: Genome-Wide Identification and Functional Verification of Core Berberine Bridge-like Enzyme Genes for Benzylisoquinoline Alkaloid Biosynthesis in Corydalis saxicola Bunting
Source: Int J Mol Sci. 2026 Jul 27;27(15):6708. doi: 10.3390/ijms27156708 (PMC13467496; doi:10.3390/ijms27156708)
Supplement: Supplementary file 1 [file ijms-27-06708-s001.zip › ijms-4390085-supplementary.pdf]

Supplementary Materials

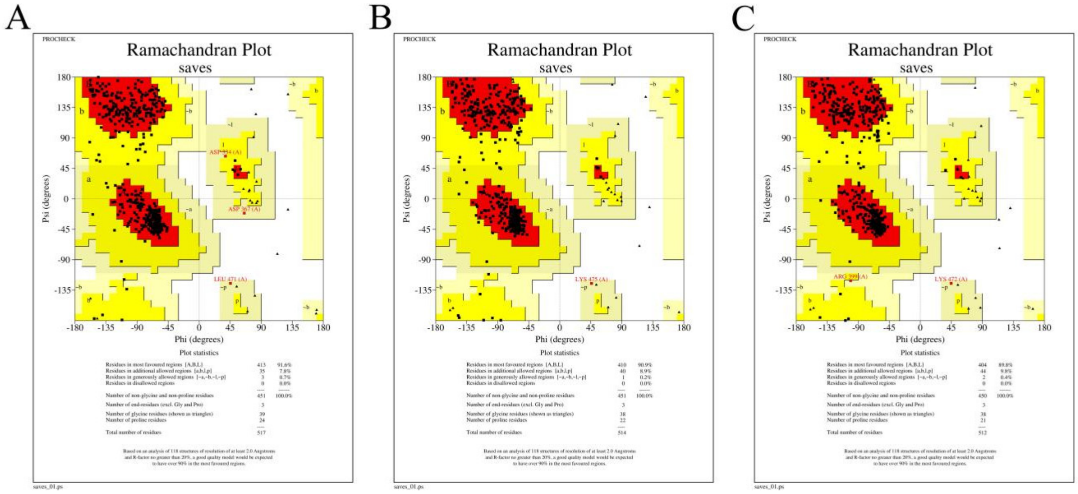

Supplementary Figure S1. Ramachandran plot validation of CsBBEL protein models used for molecular docking. A, B, and C represent the PROCHECK-generated Ramachandran plots of CsBBEL3, CsBBEL16, and CsBBEL17 protein models, respectively. All three models have over 90% of residues located in the most favoured regions, with no residues in disallowed regions.

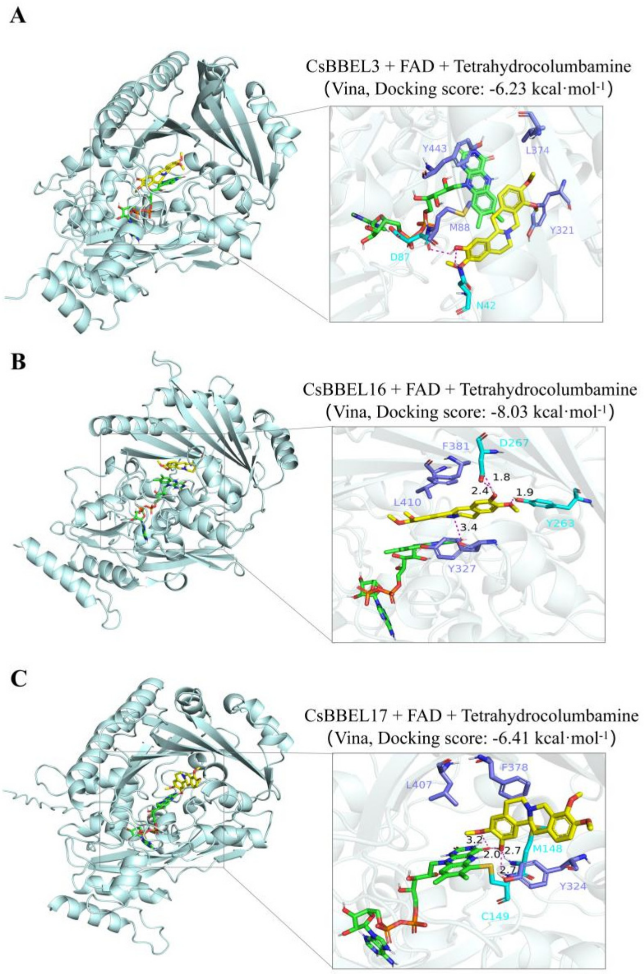

Supplementary Figure S2. Molecular docking of core CsBBEL proteins with FAD and tetrahydrocolumbamine in C. saxicola. Panels A, B, and C represent the docking results of CsBBEL3, CsBBEL16, and CsBBEL17 with tetrahydrocolumbamine, respectively.

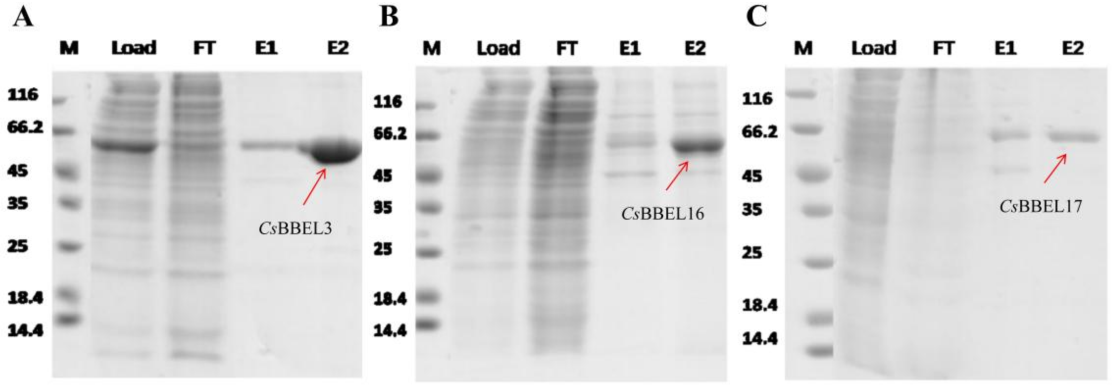

Supplementary Figure S3. SDS-PAGE analysis of recombinant CsBBEL3, CsBBEL16 and CsBBEL17 proteins expressed in Sf9 insect cells and purified via Ni-NTA affinity chromatography. (A) CsBBEL3; (B) CsBBEL16; (C) CsBBEL17. Lane M: Protein molecular weight marker; Load: Total cell lysate; FT: Flow-through fraction; E1: Low-concentration imidazole elution fraction; E2: High-concentration imidazole elution fraction (imidazole concentration in E2 > E1).

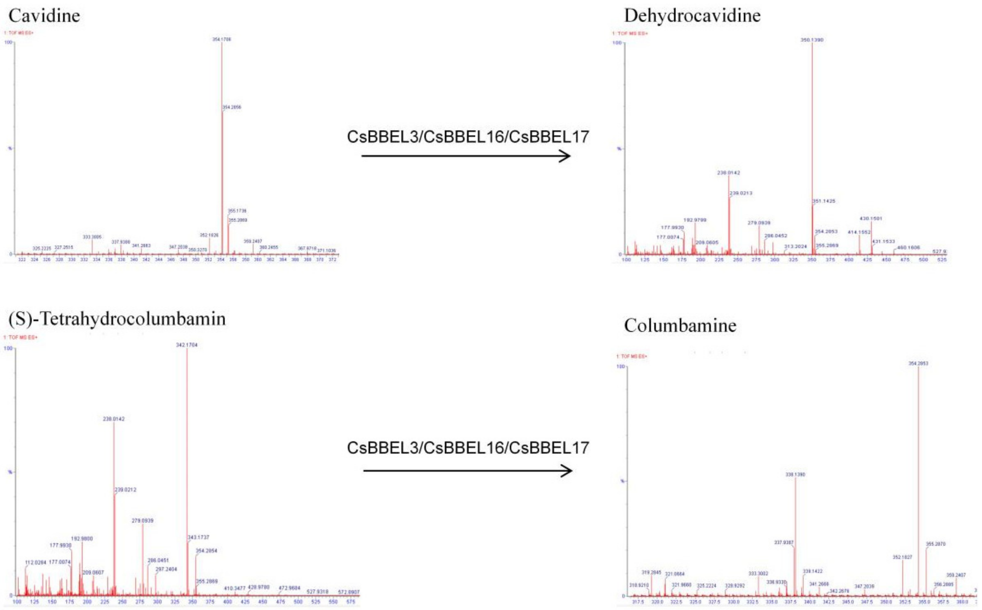

Supplementary Figure S4. The LC-MS analysis of catalytic reactions of CsBBEL3, CsBBEL16 and CsBBEL17 using cavidine and tetrahydrocolumbamine as substrates to produce dehydrocavidine and columbamine, respectively.
